# Successful Endovascular Treatment for Middle Cerebral Artery Occlusion Caused by the Thrombus Formation in the Pulmonary Vein Stump Following Left Upper Lung Lobectomy

**DOI:** 10.7759/cureus.17150

**Published:** 2021-08-13

**Authors:** Eri Shiozaki, Yoichi Morofuji, Ichiro Kawahara, Tsutomu Tagawa, Keisuke Tsutsumi

**Affiliations:** 1 Neurosurgery, National Nagasaki Medical Center, Omura, JPN; 2 Thoracic Surgery, National Nagasaki Medical Center, Omura, JPN

**Keywords:** case report, cerebral infarction, lung lobectomy, pulmonary vein stump, endovascular thrombectomy, perioperative stroke

## Abstract

Thrombus formation in the pulmonary vein (PV) stump after lung resection can cause rare cases of cerebral infarction. These infarctions can result in embolism and ischemia in the relatively large intracranial vessels, severely impacting the quality of life (QOL) of these patients. We performed endovascular thrombectomy successfully for this rare complication after lung lobectomy. A 73-year-old woman with paroxysmal atrial fibrillation (AF) suffered from sudden left complete hemiplegia 19 days after undergoing a left upper lung lobectomy (LUL). Magnetic resonance imaging (MRI) showed middle cerebral artery occlusion. Her left hemiplegia improved after the endovascular thrombectomy. Cardiogenic embolism was first suspected, but contrast-enhanced computed tomography (CECT) showed thrombus formation in the PV stump. We continued anticoagulant therapy, and the thrombus resolved completely two months after the stroke. Our patient had a relatively good outcome due to the immediate reperfusion of the affected area. This embolic source may be overlooked because AF frequently occurs after thoracic surgeries. Care should be taken during the postoperative phase to avoid overlooking these emboli. All thoracic surgeons should be informed about mechanical thrombectomy as an effective treatment for postoperative cerebral infarction.

## Introduction

Atrial fibrillation (AF) is a common complication that occurs after lung resection and sometimes causes systemic embolism [1, 2]. However, cerebral infarction due to thrombus formation in the pulmonary vein (PV) stump is rare [3, 4]. Herein we report a case of successful endovascular treatment for middle cerebral artery (MCA) occlusion due to the thrombus formation in the PV stump following a left upper lung lobectomy (LUL).

## Case presentation

The patient was a 73-year-old woman with hypertension and paroxysmal AF (CHADS2 score: 1). She was not prescribed any anticoagulants but was controlled by antiarrhythmic medications and had not reported tachycardia for a few years. She underwent LUL via video-assisted thoracic surgery for lung adenocarcinoma (pT1bN0M0, stage IA2). The postoperative course was uneventful except for paroxysmal AF, which continued for three days. The patient was then transferred to another hospital.

**Figure 1:**
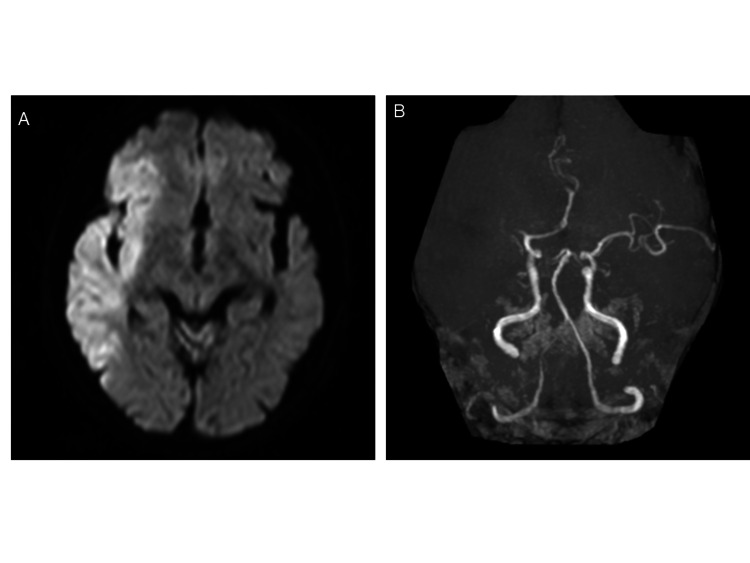
MRI obtained on initial patient admission. (A) Diffusion-weighted imaging revealed an acute ischemic stroke in close proximity of the right middle cerebral artery. DWI- Alberta Stroke Program Early CT Score was 5. (B) Magnetic resonance angiography showed a right M1 occlusion.

The patient was transferred to our hospital for sudden left complete hemiplegia 19 days after the surgery. National Institutes of Health Stroke Scale 17 and electrocardiography showed AF. Magnetic resonance imaging (MRI) taken 160 minutes after the onset showed acute infarction in the right MCA region and the right M1 occlusion (Figure 1).

**Figure 2:**
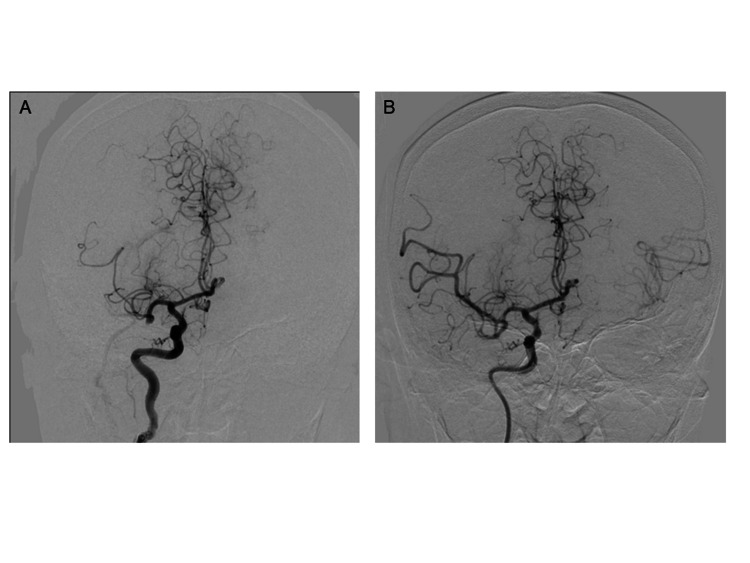
Pre and postoperative angiography. (A) Preoperative imaging showed a right M1 occlusion. (B) Thrombolysis in cerebral infarction 2b (TICI2b) reperfusion was obtained after urgent thrombectomy.

There was an MR angiography-diffusion-weighted imaging mismatch. Despite the relatively large size of the infarct area, her eloquent area still remained. Therefore, we performed endovascular thrombectomy using a stent retriever and a red thrombus was retrieved. Thrombolysis in cerebral infarction 2b reperfusion was obtained with a single pass in 28 minutes after the puncture (Figure 2).

**Figure 3:**
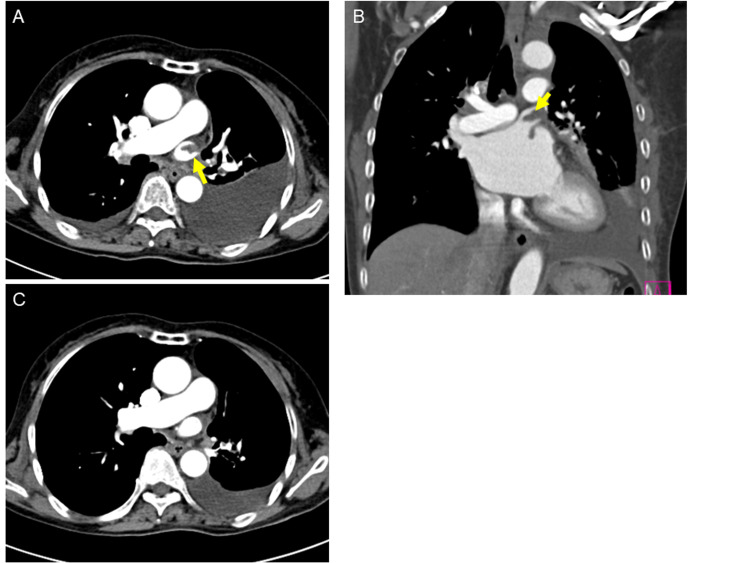
Contrast-enhanced computed tomography (CECT). (A, B) Arrows indicate thrombus formation in the left superior pulmonary vein stump on CECT seven days after onset. (C) The thrombus dissipated two months after the cerebral infarction.

The patient’s left hemiplegia improved after reperfusion. Cardiogenic embolism was first suspected, and then we started the patient on unfractionated heparin. The transthoracic echocardiogram showed no abnormality, but contrast-enhanced computed tomography (CECT) showed thrombus formation in the left superior PV stump six days after the thrombectomy (Figure 3 - A & B). Bilateral acute renal infarction was also detected on CECT, and the thrombus was thought to have caused a systematic embolism. We continued anticoagulants and opted to change from heparin to apixaban 10 days after the admission for both AF and the thrombus formation in the PV stump. The thrombus ultimately resolved two months after the initial admission (Figure 3 - C) and modified Rankin Scale 3 was achieved six months after the intervention.

## Discussion

Thrombus formation in the PV stump is a rare embolic source of cerebral infarction [3, 4] and has recently been identified as a complication that follows lung resection and could cause systemic embolism [3-7]. The thrombus formation of the PV stump occurs in 3.3% to 3.6% of patients after lung lobectomy and 13.5% to 17.9% among patients that had undergone LUL [5, 6]. The turbulent blood flow in the PV stump is thought to contribute to thrombus development [3, 5-8]. LUL is considered a risk factor for cerebral infarction [9, 10] although not all cases have shown thrombus formation within the PV stump [11, 12]. As LUL usually leaves a longer PV stump than other types of lobectomy, blood stasis may occur more frequently in these regions and result in thrombus formation [3, 5, 6].

**Figure 4:**
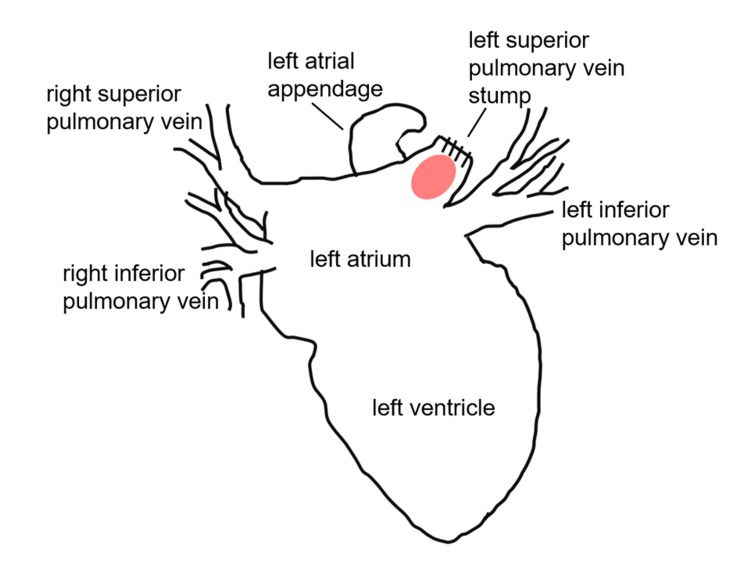
Schema of the left atrium, left atrial appendage (LAA), and the pulmonary vein (PV) stump. The pouch-like structure of the PV stump is similar to LAA.

Some reports detected spontaneous echo contrast in the left superior PV stump using intraoperative ultrasonography or transesophageal echocardiogram [8, 13]. The spontaneous echo contrast indicates turbulent flow or stasis of blood, and it is often seen in the left atrial appendage (LAA) in patients with AF [14]. LAA is the primary site for thrombus formation in AF patients, and the pouch-like morphology of the PV stump is similar to LAA (Figure 4). We expect that AF can induce thrombus formation in both the PV stump and LAA.

AF is a common complication that occurs during the early period following lung resection and sometimes causes cardiogenic embolism [1, 2]. AF occurs in 10% to 30% of the patients that undergo lung resection [2, 10]. Thrombus in the PV stump may be overlooked as cardiogenic embolism due to the frequency of the postoperative AF.

There is no consensus whether perioperative anticoagulated therapy is indispensable after lung resection to prevent the thrombus formation in the PV stump [5-6, 8-9]. In the present case, the thrombus developed despite the patient’s CHADS2 score being low and the arrhythmia controlled well during the preoperative phase. Therefore, we postulate that anticoagulated therapy is required when AF occurs after LUL, even if there are no other risk factors for stroke. As the onset of cerebral infarction following lung lobectomy varies from one day to seven years [4, 10, 13, 15], long-term follow-up and anticoagulated therapy should be continued with or without the occurrence of AF.

In previous reports, infarction following lung lobectomy tends to occur in the relatively large intracranial vessels [3, 4, 15] and can be highly critical and impact the quality of life. Our patient had a comparatively good outcome due to the immediate treatment. It is critical to consider the possibility of endovascular treatment when neurological deteriorations appear in patients following lung resection. Stroke physicians and thoracic surgeons should be aware that lung resection can be a source of emboli formation.

## Conclusions

Our patient had a comparatively good outcome due to the immediate treatment. It is critical to consider the possibility of endovascular treatment when neurological deteriorations appear in patients following lung resection. Stroke physicians and thoracic surgeons should be aware that lung resection can be a source of emboli formation.
